# Brachial Plexitis as a Rare Manifestation of Giant Cell Arteritis: A Case-Based Review

**DOI:** 10.31138/mjr.190626.rsa

**Published:** 2026-09-01

**Authors:** Noelia Cabaleiro-Raña, Evelin Cecilia Cervantes Pérez, Diego Santos-Álvarez, Carmen Álvarez-Reguera, Lucía Romar de las Heras, Sabela Fernández Aguado, María Luz Carpintero-Saiz, Susana Romero-Yuste

**Affiliations:** 1 Rheumatology Department, University Hospital Complex of Pontevedra, Pontevedra, Spain;; 2 Pathology Department, University Hospital Complex of Pontevedra, Pontevedra, Spain

**Keywords:** giant cell arteritis, brachial plexus, peripheral nervous system diseases, vasculitis, tocilizumab

## Abstract

**Background:**

Giant cell arteritis (GCA) is the most common systemic vasculitis, typically involving large and medium-sized arteries. It usually presents with cranial ischemic symptoms, although peripheral nervous system involvement may also occur. Brachial radiculoplexopathy is a particularly rare manifestation. Early recognition is essential, as prompt corticosteroid therapy generally results in substantial neurological improvement.

**Case report:**

A 69-year-old man with a history of arterial hypertension, diabetes mellitus, dyslipidaemia, hypothyroidism, and polymyalgia rheumatica presented with headache, cervical pain, and left upper limb weakness. Electrophysiological studies showed acute neurogenic changes involving the C5-C6 distribution. Temporal artery ultrasound showed a halo sign, and biopsy confirmed the diagnosis of GCA. The patient achieved full neurological recovery after four weeks of treatment with corticosteroids and tocilizumab.

**Literature review:**

A narrative review was conducted using PubMed/MEDLINE, Scopus, Embase, and Web of Science databases. Twenty-nine reported cases of brachial radiculoplexopathy associated with GCA, were identified. The mean age was 68 years, with a balanced sex distribution. Neurological in-volvement was bilateral in 59% of cases and mainly affected the C5–C6 roots. All patients had elevated in-flammatory markers, and temporal artery biopsy confirmed GCA in 97% of cases. Most patients respond-ed rapidly to corticosteroid therapy, while only a few required additional immunosuppressive treatment.

**Conclusions:**

Brachial radiculoplexopathy is a rare neurological manifestation of GCA that may occur at disease onset and pose a diagnostic challenge. Awareness is essential for timely diagnosis and treatment, as most patients respond favourably to corticosteroids. Tocilizumab may represent an effective steroid-sparing option in selected cases.

## INTRODUCTION

Acute brachial plexitis, also known as amyotrophic neuralgia or Parsonage-Turner syndrome, is a disorder characterised by acute shoulder pain with cervical and/or brachial irradiation, associated with weakness and muscle atrophy. Although it often involves the upper brachial plexus, the distribution may be variable, affecting multiple trunks or individual peripheral nerves, and may present within a radiculoplexopathy spectrum. It is usually a unilateral condition but can become bilateral in up to 25% of cases. Traditionally, it has been considered an idiopathic condition, although in a variable percentage of cases, the existence of a relevant history is identified: trauma, bacterial (Borrelia) or viral infections (flu virus, cytomegalovirus [CMV], human immunodeficiency virus [HIV]), immunisations or surgery. It has also been linked to autoimmune processes such as polyarteritis nodosa, systemic lupus erythematosus or giant cell arteritis (GCA).^1^

GCA is the most prevalent vasculitis in Europe and North America, affecting patients over 50 years of age, with a female predominance. Arterial inflammation causes luminal compromise with consequent ischaemia of the vascular territory.^2^ The involvement of the aorta and its main branches, including the subclavian and axillary arteries, may lead to upper limb ischaemia.^3^ Susceptible places for this involvement are the vascular bed of the cranial nerves, the optic nerve, the masseter muscles and the posterior circulation of the central nervous system. Peripheral neuropathy is a rare manifestation of GCA.^4^ Cases of mononeuropathy (median, radial, peroneal, or sciatic), mononeuritis multiplex, cervical radiculopathy, or brachial plexopathy have been described. Early studies suggested a relatively high frequency^5^; however, these estimates were derived from older cohorts and may overestimate its true prevalence. More recent data and contemporary cohorts suggest that peripheral neurological involvement is rare, although its exact frequency remains uncertain. The aim of this study is to provide a structured narrative review of published cases of brachial plexitis associated with giant cell arteritis, with the inclusion of an illustrative case, in order to increase clinical awareness and facilitate early diagnosis and appropriate management.

## CASE REPORT

A 69-year-old man with a personal history of arterial hypertension, diabetes mellitus, dyslipidaemia, hypothyroidism and polymyalgia rheumatica in remission presented with cervical pain, temporal headache, weakness and hypoesthesia of the left upper limb. At presentation, he reported a one-month history of cervical pain and headache with holocranial irradiation, predominantly affecting the left periocular region. Approximately two weeks after the onset of headache, he developed sudden weakness of the left upper limb, accompanied by sensory impairment. The patient was evaluated two weeks after the onset of the neurological deficit. On the same day of clinical assessment, he was admitted to hospital, and high-dose intravenous glucocorticoid therapy was initiated following vascular ultrasound findings consistent with GCA. He denied weight loss or fever.

On examination, muscle strength was assessed using the Medical Research Council (MRC) scale^6^: left shoulder abduction (grade 2/5), left elbow flexion (grade 3/5), while all other muscles were graded 5/5. There were no pyramidal signs, no ataxia, or parkinsonian features. Tactile hypoesthesia was detected on the inner surface of the left upper limb, with preserved deep tendon reflexes. General examination was otherwise unremarkable.

**Figure 1.**
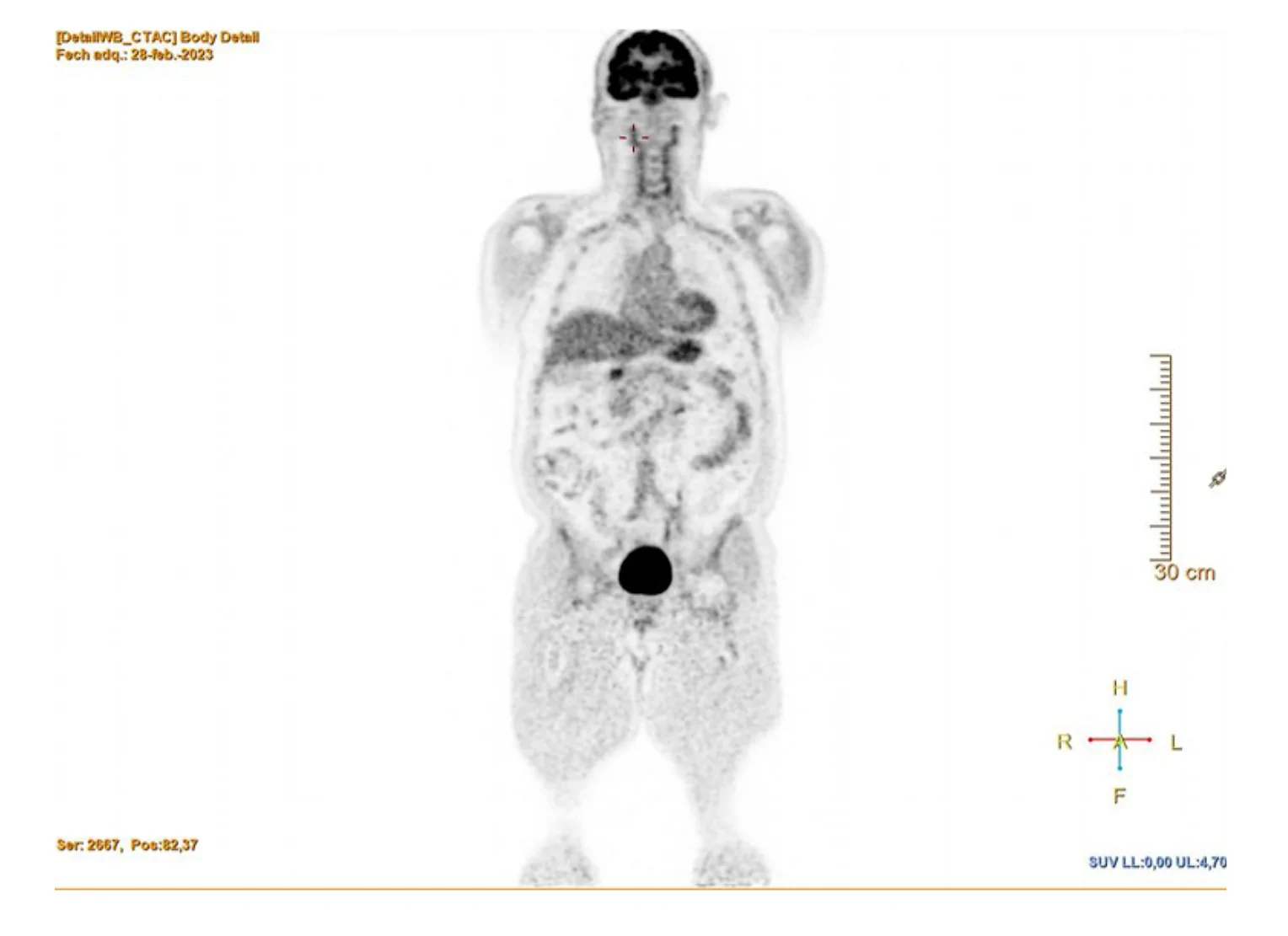
Whole-body positron emission tomography (PET) scan showing increased 18F-fluorodeoxyglucose uptake following the vascular path of the carotid arteries, vertebral arteries, brachiocephalic trunk, subclavian arteries, aortic arch, descending thoracic aorta, and abdominal aorta.

**Figure 2.**
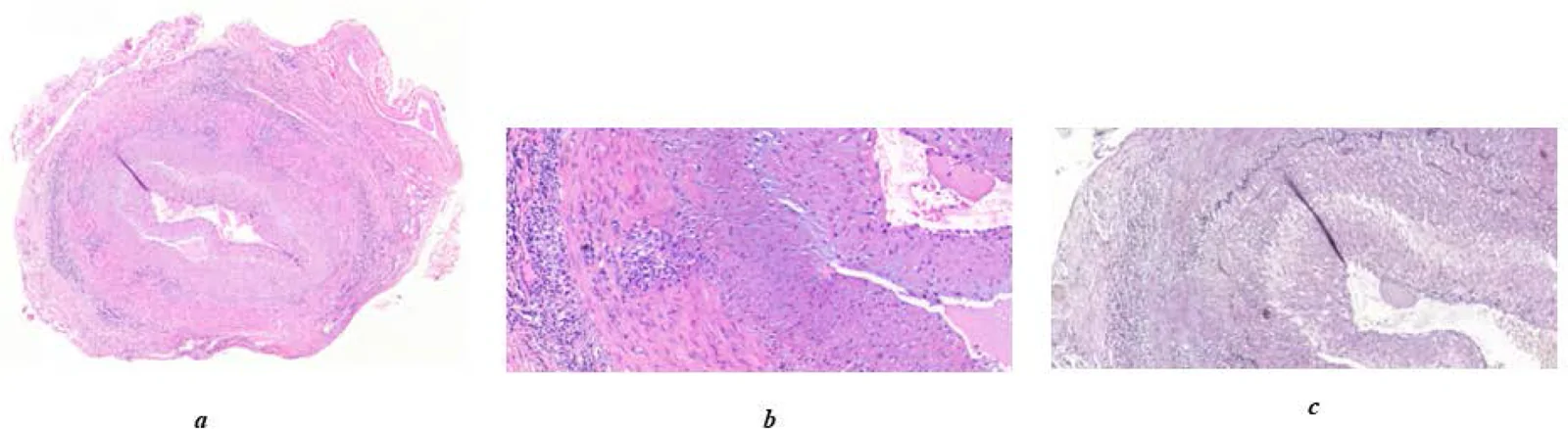
Histopathological findings of temporal artery biopsy. (a) Transmural inflammatory infiltrate with disruption of the internal elastic lamina. (b) Presence of multinucleated giant cells within the arterial wall. (c) Luminal narrowing secondary to intimal hyperplasia.

Laboratory tests revealed elevated inflammatory markers, with a C-reactive protein level of 10.8 mg/dL and an erythrocyte sedimentation rate of 58 mm/h. Creatine kinase, liver enzymes, and thyroid function tests were normal. Serological testing for infectious causes and autoimmune antibodies was negative. Electrophysiological studies demonstrated acute neurogenic changes predominantly involving the left C5– C6 distribution. Although these findings may overlap with cervical radiculopathy, the absence of structural abnormalities on cervical spine and brachial plexus imaging, together with the overall clinical context, supported a diagnosis within the brachial radiculoplexopathy spectrum. Magnetic resonance imaging of the brain, cervical spine, and brachial plexus showed no abnormalities. Temporal artery ultrasound revealed a halo sign involving the left temporal artery and whole-body positron emission tomography (PET) scan demonstrated increased 18F-fluorodeoxyglucose uptake in both cranial and extracranial arteries (**Figure 1**). GCA was also verified by temporal artery biopsy (**Figure 2**). Treatment was initiated with intravenous methylprednisolone pulses (1 g/day for three days), followed by oral prednisone (40 mg/day, tapering regimen) and acetylsalicylic acid (100 mg/day). Given the patient’s diabetes mellitus and the risk of glucocorticoid-related adverse effects, weekly subcutaneous tocilizumab (162 mg) was added. Complete recovery of left upper limb function was achieved four weeks after initiation of therapy, with progressive normalisation of inflammatory markers.

## MATERIALS AND METHODS

A narrative review of the literature was performed to identify published cases of brachial plexitis associated with giant cell arteritis. The search was conducted in PubMed/MEDLINE, Scopus, Embase, and Web of Science databases, covering publications from January 1976 to July 2025. The following search terms were used: (“brachial plexitis” OR “brachial plexopathy” OR “Parsonage–Turner syndrome” OR “neuropathy”) AND (“giant cell arteritis” OR “temporal arteritis” OR “Horton’s disease”).

Additional relevant publications were identified through manual screening of the reference lists of selected articles. Case reports and case series describing brachial plexus involvement attributed to GCA were included. Only articles written in English, Spanish, French, Italian, or Portuguese were considered. Reviews and editorials were excluded. Conference abstracts and reports were included only when sufficient clinical information was available to support the diagnosis of GCA.

The included cases were analysed descriptively, focusing on demographic characteristics, clinical presentation, diagnostic findings, treatment strategies, and outcomes.

This study was conducted in accordance with the CABARET recommendations from the EQUATOR Network for case-based reviews.

## RESULTS

Our literature review, including the present illustrative case, identified 29 reported instances of brachial plexitis in the context of GCA, as summarised in **Table 1**. The mean age at presentation was 68 ± 7.5 years, with no clear sex predominance (14 women and 15 men). Acute-phase reactants (CRP and/or ESR) were available for 27 of the 29 cases, and all showed elevated values (CRP > 0.5 mg/dl and/or ESR > 19 mm/h). ESR was greater than 50mm/h in 75% of cases, reaching levels greater than or equal to 100 mm/h in 34.5%, while CRP was elevated in all patients in whom it was quantified. Regarding clinical manifestations, headache (68.6%), neck pain (55.2%), and shoulder pain (41.4%) were the most frequent symptoms. Classic cranial features such as scalp tenderness and jaw claudication were less common, affecting 17.2% and 20.7% of cases, respectively. Constitutional symptoms were noted in 41.4%, and low-grade fever in 34.5%. Five patients (17.2%) had other neurological manifestations, including anterior ischemic optic neuropathy (AION). Temporal artery abnormalities on physical examination were reported in 31% of cases.

**Table 1.** Clinical characteristics of reported cases of brachial plexitis associated with giant cell arteritis.

**Author, Year, Country**	**Sex**	**Age**	**APR**		**CLINICAL FEATURES**													
			**CRP**	**ESR**	**Headache**	**Neck pain**	**Shoulder pain**	**Scalp tenderness**	**Jaw claudication**	**Const. Synd.**	**Fever**	**AION**	**Other**
Sánchez (1983), Spain^7^	F	73	NA	125	No	Yes	Yes	No	No	Yes	Yes	No	No
Shapiro (1983), USA^8^	F	64	NA	55	Yes	No	No	No	No	No	No	No	No
Nesher (1987), Israel^9^	M	60	NA	108	No	Yes	No	No	No	No	No	No	No
	F	63	NA	61	Yes	No	Yes	No	No	No	No	No	No
Dierckx (1990), Belgium^10^	F	73	1.8	121	Yes	No	Yes	No	No	Yes	Yes	No	No
Balquet (1991), France^11^	M	70	NA	113	No	Yes	No	No	Yes	Yes	Yes	Yes	No
	F	64	NA	100	Yes	Yes	No	No	No	Yes	Yes	No	No
Hughes (1994), Wales^12^	M	57	NA	90	Yes	Yes	Yes	No	No	No	No	No	Paralysis III c.n (L)
Rivest (1995), Canada^13^	F	53	NA	83	No	Yes	Yes	No	No	Yes	No	No	Pharyngodynia, Facial pain (L)
Mota (1995), Spain^14^	M	66	18.2	85	No	Yes	Yes	No	No	Yes	Yes	No	No
Gatfosse (1996), France^15^	M	83	NA	41	Yes	Yes	No	Yes	Yes	Yes	Yes	No	No
Benharrats (1997), France^16^	F	66	17.6	100	Yes	Yes	No	No	No	No	No	No	No
Silva (1998), Canada^17^	M	72	NA	49	No	Yes	Yes	No	No	No	No	No	Paralysis VI c.n (L)
Burton (1999), United Kingdom^18^	M	69	NA	25	No	No	No	No	No	No	No	No	Dyspnea
Dananchet (2002), France^19^	F	73	NA	88	Yes	Yes	Yes	Yes	Yes	Yes	No	No	No
Soubrier (2002), France^20^	F	75	20.9	116	Yes	No	No	No	No	No	No	No	No
Chowdhry (2002), USA^21^	M	60	NA	140	No	Yes	No	No	No	No	Yes	No	No
Saidha (2005), Ireland^22^	M	59	NA	63	Yes	No	Yes	Yes	No	No	No	No	Aortic dissection
Pfadenhauer (2007), Germany^23^	F	63	9.6	88	Yes	No	Yes	Yes	Yes	No	No	No	No
	F	74	NA	NA	Yes	No	No	Yes	No	No	Yes	No	No
	M	82	NA	NA	Yes	No	No	No	No	No	No	No	No
Blaise (2005), France^24^	F	67	12.2	94	Yes	Yes	No	No	No	Yes	No	No	Alt. V1, V2 c.n (L)
Klein (2011), Germany^25^	M	79	18.1	79	Yes	No	No	No	No	Yes	No	No	No
London (2015), Belgium^26^	M	64	25	NA	No	Yes	Yes	No	No	Yes	Yes	No	No
Duval (2018), France^27^	F	85	17.6	100	No	No	No	No	No	No	No	No	Paralysis III c.n (R)
Calle López (2018), Colombia^28^	M	70	23	90	Yes	No	No	No	Yes	Yes	No	No	No
Nakabayashi (2021), Japan^29^	M	72	13.9	31	No	No	Yes	No	No	No	No	No	No
Bounia (2022), Greece^30^	F	71	5	117	No	Yes	No	No	Yes	No	Yes	No	Pharyngodynia
Cabaleiro-Raña (2024), Spain	M	69	10.8	58	Yes	Yes	No	No	No	No	No	No	No

APR: acute phase reactants; CRP: C-reactive protein; ESR: erythrocyte sedimentation rate; Const. Synd.: constitutional syndrome; AION: anterior ischemic optic neuropathy; NA: not available; MRI: magnetic resonance imaging; CTA: computed tomography angiography; DSA: digital subtraction angiography; US: ultrasound; PET: positron emission tomography; Bi: bilateral; L: left; R: right; c.n.: cranial nerve; (+): positive; (−): negative.

**Table 2.** Diagnostic findings, treatment, and outcomes of reported cases of brachial radiculoplexopathy associated with giant cell arteritis.

**Author, year, Country**	**Affected roots (side)**	**Imaging tests**	**Biopsy +**	**Response**
Sánchez (1983), Spain^7^	C5,C6 (R)	No imaging performed	Yes	Complete
Shapiro (1983), USA^8^	C5,C6 (Bi)	No imaging performed	Yes	Complete
Nesher (1987), Israel^9^	C5 (Bi)	No imaging performed	Yes	Complete
	C5 (R)	No imaging performed	Yes	Complete
Dierckx (1990), Belgium^10^	C5,C6 (Bi)	MRI (-); CTA (-)	Yes	Complete
Balquet (1991), France^11^	C5 (Bi)	US (+)	Yes	Partial
	C5.C6 (L)	US (+)	Yes	Complete
Hughes (1994), Wales^12^	C4-C6 (Bi)	MRI (-)	Yes	Complete
Rivest (1995), Canada^13^	C5 (L)	DSA (+)	Yes	Complete
Mota (1995), Spain^14^	C5-C8 (L)	MRI (-)	Yes	Complete
	C5-C6 (R)			
Gatfosse (1996), France^15^	C5,C6 (Bi)	No imaging performed	Yes	Complete
Benharrats (1997), France^16^	C5,C6 (Bi)	No imaging performed	Yes	Complete
Silva (1998), Canada^17^	C5.C6 (Bi)	No imaging performed	Yes	Complete
Burton (1999), United Kingdom^18^	C4-C8 (Bi)	MRI (-)	No	Not treated. (dead)
Dananchet (2002), France^19^	C5-C7 (L)	No imaging performed	Yes	Complete
Soubrier (2002), France^20^	C5.C6(R)	No imaging performed	Yes	Complete
Chowdhry (2002), USA^21^	C5-C7 (R)	No imaging performed	Yes	Complete
Saidha (200: ), Ireland^22^	C5-C7 (Bi)	MRI (-)	Yes	Complete
	C5,C6 (L)	US (+)	Yes	Complete
Pfadenhauer (2007), Germany^23^	C5-C7 (L)	US (+); MRI (+)	Yes	Complete
	C5-C7(L)	MRI (-)	Yes	Partial (dead)
Blaise (2005), France^24^	C5.C6 (L)	MRI (-)	Yes	Complete
Klein (2011), Germany^25^	C5,C6 (Bi)	MRI (-)	Yes	Complete
London (2016), Belgium^26^	C5,C6 (Bi)	MRI (-); PET (+)	Yes	Complete
Duval (2018), France^27^	C5,C6 (Bi)	MRI (-); PET (+)	Yes	Complete
Calle López (2018), Colombia^28^	C5,C6 (Bi)	US (-); MRI (-); PET (+)	Yes	Complete
Nakabayashi (2021), Japan^29^	C4-C6 (Bi)	US (-); PET (+)	Yes	Complete
Bounia (2022), Greece^30^	C4-C8 (Bi)	US (+); MRI (+); PET (+)	Yes	Complete
Cabaleiro- Raña (2024), Spain	C5,C6 (L)	US (+); MRI (-); PET (+)	Yes	Complete

APR: acute phase reactants; CRP: C-reactive protein; ESR: erythrocyte sedimentation rate; Const. Synd.: constitutional syndrome; AION: anterior ischemic optic neuropathy; NA: not available; MRI: magnetic resonance imaging; CTA: computed tomography angiography; DSA: digital subtraction angiography; US: ultrasound; PET: positron emission tomography; Bi: bilateral; L: left; R: right; c.n.: cranial nerve; (+): positive; (−): negative.

Brachial plexopathy was bilateral in 58.6% of cases and unilateral in 41.4%, with a slight predominance of the left side. The most affected nerve roots were C5 and C6, which had a certain degree of damage in all patients. C4 involvement was found in 4 patients, one of whom died due to respiratory failure secondary to diaphragmatic weakness. In most reported cases, brachial plexopathy occurred in association with other systemic or cranial features of GCA. However, the temporal relationship between neurological symptom onset and the diagnosis of GCA was not consistently reported across all cases.

Vascular ultrasound was performed in 8 of the 29 patients, revealing evidence of vessel involvement in 6 of them. Angio-magnetic resonance imaging was performed on 14 patients, revealing brachial plexitis changes in only 2 of them. Positron emission tomography (PET) revealed signs of vasculitis in all patients who underwent this procedure (6 of 29 patients). Finally, temporal artery (TA) biopsy was performed in all patients, which was compatible with GCA in 28 of them. All patients received moderate-to high-dose of glucocorticoids, except one diagnosed postmortem. The vast majority of patients (93%) improved only with glucocorticoid treatment. Corticosteroid-sparing treatment was administered in two patients, including the present illustrative case, both of whom were treated with tocilizumab. Overall, two deaths were reported: one related to bilateral diaphragmatic paralysis and another associated with vertebral artery involvement diagnosed postmortem.

## DISCUSSION

Brachial plexitis represents a rare but clinically relevant neurological manifestation of giant cell arteritis (GCA), which may occur at disease onset and pose a significant diagnostic challenge. Peripheral nervous system involvement in GCA is considered uncommon. While GCA predominantly affects women (female-to-male ratio of about 2:1), published cases of brachial plexitis suggest no clear sex predominance. The rarity of this presentation, together with the broad differential diagnosis of brachial plexopathies, may delay recognition and appropriate treatment if GCA is not considered early in the diagnostic process.

The pathophysiological mechanisms underlying brachial plexus involvement in GCA remain poorly understood. Several hypotheses have been proposed, including ischemic injury related to involvement of the vasa nervorum, extension of inflammation from adjacent large vessels, or immune-mediated mechanisms.^7^ However, these explanations are not specific to GCA and may also be observed in other forms of vasculitis. Therefore, the exact pathophysiological link between GCA and brachial plexopathy remains uncertain. Several reports have described bilateral or multifocal patterns, which may support a vascular mechanism rather than a compressive aetiology. Moreover, the rapid and often complete neurological recovery observed after prompt initiation of glucocorticoid therapy suggests an inflammatory and potentially reversible process, a pattern that contrasts with the typically slower and incomplete recovery seen in necrotising vasculitides or primary demyelinating disorders. More recently, the use of interleukin-6 inhibition has emerged as a potential adjunctive strategy in selected cases, particularly in patients at high risk of glucocorticoid-related adverse effects.

From a clinical perspective, recognising brachial plexopathy as a potential manifestation of GCA is important, particularly in older patients presenting with acute upper limb pain or weakness together with elevated inflammatory markers. Although most reported cases occurred in association with typical systemic or cranial features of GCA, it remains unclear whether brachial plexopathy may occasionally represent an early or even isolated manifestation. In this context, failure to consider GCA in the differential diagnosis may lead to diagnostic delay and postponement of appropriate treatment. Therefore, awareness of this rare presentation may facilitate earlier diagnosis and timely initiation of therapy, potentially improving outcomes. Importantly, this report expands the existing literature by providing an updated synthesis of published cases together with a well-characterised illustrative case, building on previously reported data. Increasing awareness of atypical neurological manifestations such as brachial radiculoplexopathy may facilitate earlier diagnosis and timely treatment, potentially improving clinical outcomes.

This study has several limitations. First, this narrative review is based on published case reports and small case series, which limits the generalisability of the findings and introduces an inherent risk of publication bias. Second, the heterogeneity of available cases, particularly regarding diagnostic criteria, treatment approaches, and outcome measures, restricts the ability to draw firm conclusions. Further observational studies would be helpful to better characterise this uncommon manifestation. Despite these limitations, this review has notable strengths. It focuses on a rare and diagnostically challenging manifestation of GCA and provides the most comprehensive synthesis to date of published cases of brachial plexitis associated with this condition. By systematically summarising clinical features, diagnostic findings, treatments, and outcomes, this work aims to enhance clinical awareness and facilitate earlier recognition and timely intervention in similar presentations.

## CONCLUSION

Involvement of the peripheral nervous system is uncommon in patients suffering from active GCA. Brachial plexopathy is a rare initial presentation of GCA, but should be considered in elderly patients presenting with varying degrees of neurological impairment and elevated inflammatory markers (CRP or ESR). Based on the findings of this narrative review, vascular ultrasound, positron emission tomography, and temporal artery biopsy appear to be the most useful complementary investigations for establishing the diagnosis. Brachial plexitis associated with GCA generally shows a favourable response to glucocorticoid therapy, particularly when treatment is initiated promptly, which may help prevent permanent neurological deficits.

## ORIGINALITY STATEMENT

This manuscript is original, has not been published previously, and is not under consideration for publication elsewhere.
